# Compromised knee internal rotation in total knee arthroplasty patients during stair climbing

**DOI:** 10.1371/journal.pone.0205492

**Published:** 2018-10-10

**Authors:** Igor Komnik, Sina David, Johannes Funken, Christine Haberer, Wolfgang Potthast, Stefan Weiss

**Affiliations:** 1 Institute of Biomechanics and Orthopaedics, German Sport University Cologne, Cologne, Nordrhein-Westfalen, Germany; 2 ARCUS Clinics Pforzheim, Pforzheim, Baden-Württemberg, Germany; University of Memphis, UNITED STATES

## Abstract

Due to the significant role of rotational properties for normal knee function, this study aimed to investigate transverse plane kinematics and kinetics in total knee arthroplasty and unicondylar knee arthroplasty patients during activities of daily living compared to a healthy control group, including stair ascent and descent. The study participants consisted of a total knee arthroplasty group including posterior cruciate retaining and posterior stabilized designs as well as a unicondylar knee arthroplasty group and a healthy control group. Three-dimensional kinematics and kinetics were captured using a Vicon system and two Kistler force plates embedded in the floor and another two in a staircase. Inverse dynamics of the lower limbs was computed in Anybody^™^ Modeling System. Transverse plane joint angles and joint moments were analyzed utilizing the statistical non-parametric mapping approach, considering the entire curve shape for statistical analysis. The patients with total knee arthroplasty exhibited significantly reduced knee internal rotation of the operated knee compared to the control group and the patients’ unimpaired limb, especially during the stair climbing tasks. Both unicondylar and total knee arthroplasty patients were found to have similar reduced internal rotation motion time series in stair descent. In conclusion, potential kinematic and kinetic benefits of unicondylar knee arthroplasty over total knee arthroplasty could not be proven in the current study. Aside from the usually mentioned reasons inducing constrained knee internal rotation in total knee arthroplasty patients, future studies should investigate to what extent co-contraction may contribute to this functional impairment in patients after knee arthroplasty surgery.

## Introduction

Three-dimensional motion analysis enables examiners to grasp pathologic joint kinematics and kinetics in patients before and after knee arthroplasty (KA) surgery. Despite functional improvements after surgery [1,2], studies showed knee mechanics during activities of daily living (ADL) in patients with total knee arthroplasty (TKA) different from unimpaired controls, particularly in the sagittal plane. This included, among other things, the *stiff knee gait pattern* that is associated with reduced knee flexion angles, range of motion and flexion moments. Conversely, other patients exhibited a flexor moment pattern associated with high flexion moments throughout the whole stance phase [3–6].

KA-patients might reveal functional deficits which are not apparent in straight walking, but during more complex and physically more challenging ADL due to the persistence of muscle strength deficits, impaired proprioception or different prosthesis types [7]. In this regard, stair climbing is one of the most frequently performed and demanding activities for KA-patients, showing 12% greater resultant force values than straight walking in the operated (op)-knee [8,9]. Conspicuous inter-individual load variations were detected in ascending and descending stairs, particularly in the transverse plane [8]. Moreover, appreciably impaired knee internal rotation angles were shown during decline walking in TKA-patients’ (op)-knee that were not apparent in straight level walking [10]. Studies demonstrated compromised knee internal rotation accompanied by increased hamstrings muscle activity during various activities in subjects with ACL-deficient knees. This functional adaptation is presumed to be a compensatory mechanism for the lack of ACL [11,12]. Besides, high congruency between the femoral component and the tibial inlay is supposed to contribute to restricted knee internal rotation [13,14]. This is particularly relevant when comparing rotational behaviour of the tibia in TKA and unicondylar knee arthroplasty (UKA) patients, since tibial inlays in UKA are usually flat. Thus, the rotational behaviour should not be affected by the congruency between the femoral component and tibial insert in UKA.

However, the literature primarily focuses on straight level walking in the field of motion analysis with KA-patients, neglecting in a large part non-sagittal plane biomechanics [15]. Aside from the axial forces acting on the tibial components, less is known about the contribution of forces acting in the transverse plane to tibial plateau migration. Torsional loads are presumed to transmit frictional forces to the bone-implant interface, which can lead to early migration and ultimately develop into component loosening [13,14]. Moreover, the results of Johnson et al. [16] emphasize the importance of transverse plane knee rotation in terms of its highly significant contribution to wear of the ultra-high molecular weight polyethylene in simulated gait cycles.

In the context of statistical evaluation of biomechanical parameters, researchers usually extract discrete values from averaged time series data at certain gait cycle events for statistical hypothesis testing. However, these directional hypothesis tests mostly consider different peak values of given curve shapes, neglecting potentially relevant information. In this regard, in the field of gait analysis with KA-patients several authors used more comprehensive statistical methods like the principal component analysis [17–19]. This statistical approach covers the essential characteristics of the entire curve shape, whereby it represents rather a data reduction technique and less a hypothesis testing technique [20,21]. Statistical parametric mapping (SPM), which is based on the random field theory, represents a further more complex method in order to analyze statistically continuous n-dimensional datasets for regionally specific effects [22]. In the recent years, SPM was widely used primarily in the field of neuroimaging [23] and Pataky and colleagues introduced this method in the field of biomechanics. Especially, when analyzing pathological locomotion data, acquired from KA-patients, potentially considerable regions may be overlooked due to subjective selection of discrete values from curve shapes [20]. SPM provides a non-directional statistical hypothesis testing that is necessary if one is aims to investigate the influence of e.g. different types of knee endoprosthesis or locomotion task on patients’ kinematics and kinetics.

A better understanding of how far neuromuscular challenging ADL are capable to expose abnormal transverse plane knee biomechanics, allows conclusions to be drawn on the influence of prosthetic design on knee function. Hence, the aim of the current retrospective case control study was to investigate transverse plane knee kinematics and kinetics in patients with TKA and UKA surgery during straight walking and stair climbing, using SPM for the statistical analysis of time series. It was hypothesized that only the TKA-group would exhibit differences in kinematics and kinetics when compared to a control group (CG), primarily during stair ascent and stair descent.

## Methods

### Ethics statement

All subjects signed a written informed consent prior to data collection. The Ethics Committee of the German Sport University Cologne approved the project to be conducted in the presented form (approval number: 025/2014).

### Participants

The measurements were conducted in the laboratory of the Institute of Biomechanics and Orthopaedics (German Sport University Cologne) and in the facilities of the Arcus Clinics Pforzheim (Germany). All subjects with primary unilateral UKA and TKA for knee degenerative osteoarthritis were recruited from ARCUS Clinics Pforzheim. Initially, 271 TKA and 110 UKA patients were considered suitable for examination based on the exclusion criteria described below. Eighty-four KA-patients consented to participate in the current study. After a telephone interview 38 subjects had to be excluded. Ultimately, 24 patients formed the TKA-group and 22 subjects represented the UKA group. Due to marker tracking issues two TKA and two UKA patients had to be excluded from further analysis during stair ascent and descent. Another UKA subject had to be excluded from the evaluation of the stair descent trials due to pain in the op knee. No force plates were installed in the ground during the gait measurements in the Arcus Clinics Pforzheim. Therefore, joint moments were calculated for only eleven subjects of the TKA-group and 13 subjects of the UKA-group during the gait trials (sample sizes for kinetic results during gait are indicated in brackets, see Table 1). A healthy age matched CG consisted of 13 subjects who reported no knee pain and functional impairments for a period of one year prior to testing.

**Table 1:** Subjects' characteristics and spatial-temporal parameters.

	**Group**		**Mass [kg]**	**Height [m]**	**BMI [kg/m**^**2**^**]**	**Age [years]**	**♂/♀**	**Op knee**
	CG		67.5 ± 11.9	1.68 ± 0.1	23.8 ± 3.2	55.6 ± 4	7/6	-
	TKA		80 ± 9.7*^CG-TKA^	1.72 ± 0.1	27 ± 2.2*^CG-TKA^	60.1 ± 7	13/11	9 left/15 right
	UKA		79.8 ± 10.9*^CG-UKA^	1.71 ± 0.1	27.4 ± 2.6*^CG-UKA^	61.1 ± 6.2	13 /9	12 left/10 right
**Task**		**n**	**Velocity [m/s]**	**Contact time [s]**	**Step length [cm]**	**Step width [cm]**		
Walking	CG	13	1.4 ± 0.14	0.67 ± 0.05	39.2 ± 3.3	4.9 ± 1.5		
	TKA	22 (11)	1.4 ± 0.02	0.64 ± 0.05	38.7 ± 2.1	4.7 ± 1.2		
	UKA	20 (13)	1.4 ± 0.03	0.65 ± 0.05	38.5 ± 2	5 ± 1.5		
Stair descent	CG	13	0.58 ± 0.04	0.70 ± 0.06				
	TKA	22	0.57 ± 0.06	0.76 ± 0.09				
	UKA	19	0.53 ± 0.04*^CG-UKA^	0.90 ± 0.28*^CG-UKA^				
Stair ascent	CG	13	0.57 ± 0.06	0.80 ± 0.06				
	TKA	22	0.54 ± 0.04	0.88 ± 0.07*^CG-TKA^				
	UKA	20	0.53 ± 0.07	0.97 ± 0.27*^CG-UKA^				

*Indicates a significant difference between corresponding groups.

Exclusion criteria were (1) further joint arthroplasties, (2) musculoskeletal impairments that affected ADL, (3) pain or functional impairment in the non-op knee, (4) body mass index (BMI) greater than 31 kg/m^2^, (5) uncontrolled high blood pressure and cardiovascular events, (6) neurological disorders, (7) rheumatic diseases, (8) limb-valgus deformity greater than 7° and limb-varus deformity greater than 4°, (9) knee flexion contracture greater than 5° and (10) time of surgery less than one year or more than two and a half years prior to the examination. In order to reduce soft tissue artifacts, particular attention was given to exclude obese subjects (see Table 1).

All UKA patients received a medial cemented endoprosthesis (Unicompartmental High Flex Knee System, Zimmer, Warsaw, USA). Seventeen TKA patients received a cemented posterior stabilized endoprosthesis (TKA-PS) (SIGMA, DePuy Synthes, Warsaw, USA) and seven patients received a cemented posterior cruciate ligament preserved endoprosthesis (TKA-CR) (Genesis II, Smith and Nephew, Memphis, USA).

A minimally invasive approach was used in all UKA patients and a standard medial parapatellar approach in all TKA patients. Motion analysis was conducted on average 1.8 ± 0.4 years post-surgery (range: one year—two years and four months).

### Data acquisition and experimental procedure

**Fig 1:**
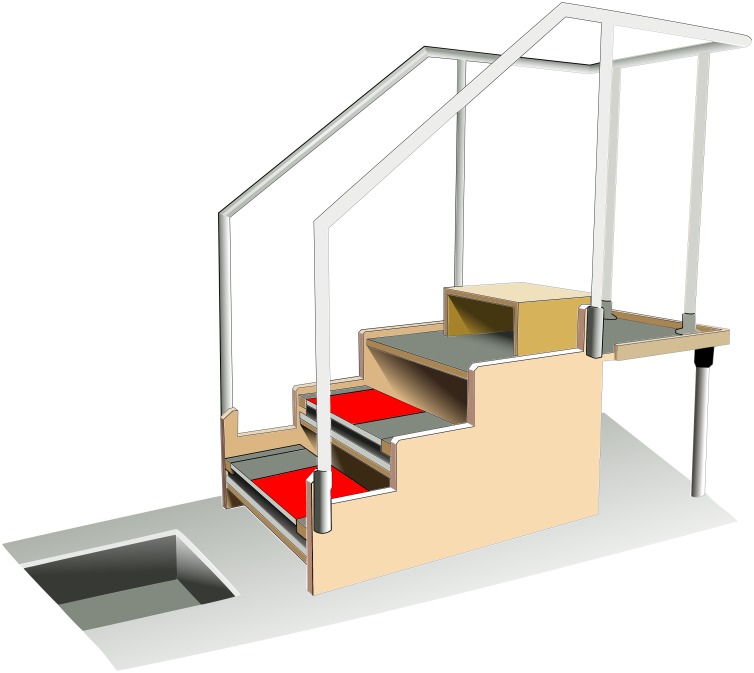
Illustration of the force plate mounted staircase. Steps with integrated load cells are depicted in red.

Kinematic data was obtained by means of a 10-camera 3D motion analysis system sampled at 100 Hz (VICON MXF40, Vicon Motion Systems Ltd, Oxford, UK). Simultaneously, ground reaction forces were collected at 1000 Hz using four Kistler force plates (Kistler Instrumente AG, Winterthur, CH). Two force plates were embedded in the floor of the Institute of Biomechanics and Orthopaedics (0.6*0.9 m, width*length). Two force plates, each (0.4*0.3 m, width*length) was mounted in the second and third step of a staircase consisting of overall four steps (0.2*0.3*0.74 m, height*depth*width, Fig 1).

In order to create a lower-limb model comprised of nine rigid segments, 28 spherical, retroreflective markers were attached bilaterally to the anterior and posterior superior iliac spines, greater trochanters, lateral and medial condyles of the femurs, tibias, lateral and medial malleoli, heads of the first and fifth metatarsals, second proximal phalanges, lateral, medial and backside of the calcanei. Data collection started with a standing reference trial. Subsequently, the CG, which was tested before both KA groups, practiced straight walking on a 15-m walkway. The captured valid trials were within 5% deviation of the practiced speed. After several practice trials TKA and UKA subjects performed straight walking at the CG’s natural average gait velocity of 1.4 m/s ± 5%. Finally, all subjects negotiated stair ascent and descent in a step-over-step manner without the use of rails at their natural velocity in order to avoid impairments of subjects' habitual stair climbing pattern. The velocity of level walking was controlled by using a time-gate system (WEKO, Weitmann & Konrad GmbH & Co KG., DE) and subsequently calculated by means of the center of mass longitudinal displacement. A trial was valid if the locomotion task was performed in a natural manner. The subjects were asked to tell the examiners if they needed a rest or felt any discomfort or pain during the measurements.

### Data analysis

Kinematic and kinetic raw data were filtered by means of a recursive, second-order Butterworth low-pass filter at a 6-Hz cutoff frequency. Anybody^™^ Modeling System (AnyBody Technology, Aalborg, DK) was used to perform lower-limb inverse dynamics according to the anatomical landmark scaled musculoskeletal model presented by Lund et al. [24]. Standing reference trials were recorded for each subject to create a stick-figure model that was used to scale a cadaver dataset into subject-specific joint parameters. The defined knee joint coordinate system was based on Pennock and Clark [25]. The computed angles from standing reference trials were subtracted from the appropriate dynamic captured trials. The modeled head and trunk were driven by the pelvis markers. In order to determine inertial properties more accurately, subjects’ whole-body anthropometrics were measured to adjust the inverse dynamic model. The mass of a segment was assumed to be the product of the volume of a frustum and the segment’s density [26]. The knee joint was modeled as a spherical joint including three degrees of freedom, which were constrained using Anybody^™^’s Force-Dependent Kinematics method [27]. Kinematic and kinetic data were time-normalized to the stand phase. The average of five to six valid trials was used for the statistical analysis. KA patients’ op-limbs were compared with the right limbs of the CG as well as with their own non-op limbs. Further data processing was conducted with custom-built Matlab (2016b) routines (The MathWorks, Natick, USA). Transverse plane knee biomechanics were assessed by means of 3D joint angles and joint moments, considering the entire time series of the stance phase. Joint moments are presented as external moments normalized to each subject’s mass and height. The step length and width were normalized to each subject’s height.

### Statistical analysis

#### Subject’s characteristics and time-distance parameters

The Shapiro-Wilk Test was used for normality testing. If the variables were normally distributed, univariate ANOVA was used to examine group differences. Individual between-group differences were clarified by means of a post hoc Tukey or Games-Howell test if the condition of homogeneity of variances was not fulfilled. If variables were not normally distributed, the non-parametric Kruskal-Wallis Test was performed to detect between-group differences. The alpha level was set at 0.05 to detect statistically significant differences. Statistical analysis was performed with in Matlab (2016b) implemented Statistics Toolbox.

#### SnPM analysis

Due to the relative small sample size in each group the statistical non-parametric mapping (SnPM) approach was used to test the null hypothesis by statistically analyzing the entire knee internal rotation angle and moment time series proposed by Nichols and Holmes [28]. Both, the parametric and non-parametric methods describe the behavior of random data, but SnMP is based on probability density functions (PDF) irrespective of data distribution, which is in contrast to the parametric approach, assuming normal Gaussian distribution. The non-parametric PDF approach includes the label permutation procedure. This implies labeling the original data according to the appropriate group and subsequently a randomly permutation of the labels. Univariate ANOVA F statistics were calculated in the form of SnPM{F} trajectories for each permutation. In order to determine the primary permutation PDF, maximum F values from each SnPM{F} trajectory were extracted. By means of the primary permutation PDF, a critical F value (critical threshold) was calculated at which only α = 5% of all permutations exceeds the critical threshold. If the original SnPM{F} exceeds the critical threshold, the null hypothesis can be rejected. The maximum suprathreshold cluster integral was extracted from each SnPM{F} trajectory to build a secondary permutation PDF and therewith verifying the rejection decision. Ultimately, the specific cluster-level p values were calculated from the secondary permutation PDF [29].

If the SnPM{F} result was significant (α<0.05) and the critical threshold was exceeded, a post-hoc analysis was conducted. The post hoc testing implied two-sample t-tests (SnPM{t}) continuums conducted on all three participated groups. The Bonferroni correction was used to adjust the critical p values for multiple comparisons. The resulting critical p value was 0.017. The SnPM evaluation was performed using the open-source spm1d package (v.M0.4, www.spm1d.org) in Matlab (R2016b).

In analogy to the univariate SnPM {F} continuums or trajectories respectively, paired SnPM{t} continuums were computed in order to evaluate symmetry between the op knee and the non-op knee of both KA-groups’ internal rotation motion and moment time series.

## Results

### Subject’s characteristics and time-distance parameters

The TKA and the UKA subjects had an 18% and 16% higher body mass accompanied by 14% greater BMI values in comparison to the CG (CG vs TKA: p = 0.0062, CG vs UKA: p = 0.0037, TKA vs UKA: p = 0.9704). Further characteristics of all subjects are presented in Table 1 as mean values ± standard deviation. In combination with the slower completed stair descent task, the UKA-group revealed statistically significant 22% prolonged foot contact time of the op knee in comparison to the CG (p = 0.003). Foot contact time during stair negotiation significantly increased for both KA groups (by 9% in the TKA-group (p = 0.016) and 18% in the UKA group (p = 0.017)).

### SnPM analysis

#### Comparison between posterior cruciate preserving and substituting TKA

No differences were detected between the TKA-CR and TKA-PS group with regard to sagittal and transverse plane rotations irrespective of the locomotion task (see supporting information). Hence, the two TKA-PS and -CR groups were combined into a single TKA group.

#### Group comparison

**Fig 2:**
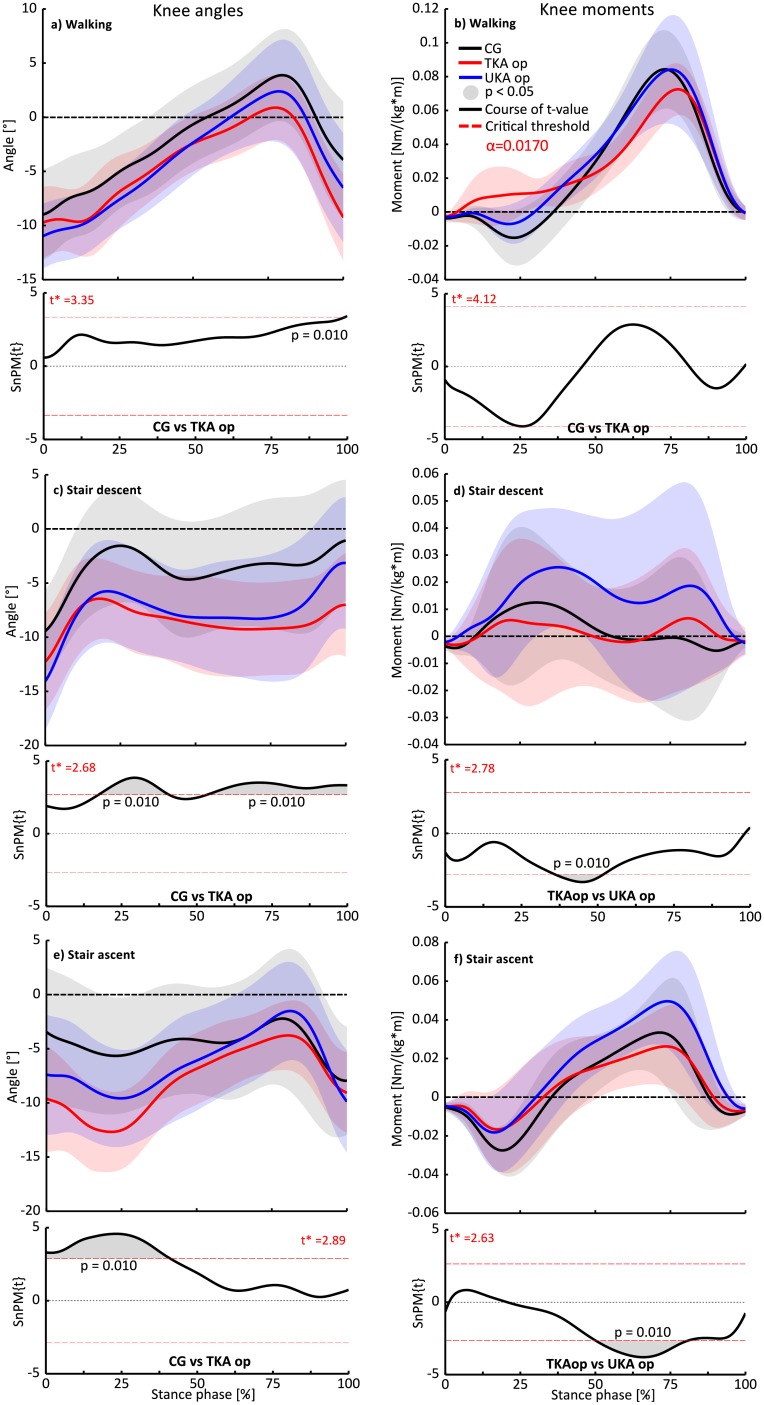
Transverse plane knee kinematics and kinetics group comparisons during walking (a, b), stair descent (c, d) and stair ascent (e, f). The time series with positive values indicate internal rotation angles or moments respectively and are presented as mean curves ± standard deviations (SD, shaded areas). SnPM-{t/F} trajectories (post-hoc comparisons between groups) are illustrated below each subfigure. If a {t/F}-trajectory exceeds the appropriate critical threshold (α = 0.017), the null-hypothesis can be rejected. The width of the exceeded threshold characterizes the temporal extent of the null-hypothesis (suprathreshold clusters illustrated by grey shaded areas underneath the SnPM-{t/F} trajectories).

Both KA-groups showed similar knee internal rotation curve progressions during all investigated locomotion tasks. The SnPM post hoc analysis illustrated in Fig 2(c) and 2(e) exposed that the knee internal rotation angle datasets of the TKA-group were significantly reduced compared to the corresponding time series of the CG in walking, stair descent and ascent. Two suprathreshold clusters exceeded the critical threshold t-value of 2.68 in stair descent task shortly after the weight acceptance (17–41%, p = 0.01) and mid-stance phase (53–100%, p = 0.01), which clarifies that the knee internal rotation motion was compromised during the major part of the stance phase (Fig 2e). Similar to the previously mentioned statistically significant regions, the stair ascent task exhibited significantly more external rotated time series within 0–40% of stance phase in the TKA-group compared to the CG. This was indicated by a suprathreshold cluster that exceeded the critical threshold t-value of 2.89 (p = 0.01).

The SnPM analysis of the knee internal rotation moment time series detected statistically significant differences between the TKA- and UKA-group considering the entire stance phase during stair descent and ascent (Fig 2d and 2f). A suprathreshold cluster exceeded the critical threshold t-value of 2.78 during 36–52% of stance in stair descent and the t-value of 2.63 during 50–81% of stance in stair ascent. However, distinct inter-individual moment variations were found in the stair descent task in each group, indicated by high standard deviations (Fig 2d).

In contrast to the CG and UKA-group, a different internal rotation moment pattern was apparent within approx. 5–40% of stance in the TKA-group during walking, albeit the course of the t-value narrowly missed the critical threshold of 4.12 (TKA-group vs CG). Hence, the null hypothesis could not be rejected. The highest mean internal rotation moment values were found in walking (CG: 0.084 Nm/(kg*m) ± 0.02, TKA op: 0.073 Nm/(kg*m) ± 0.01, UKA op: 0.084 Nm/(kg*m) ± 0.04).

**Fig 3:**
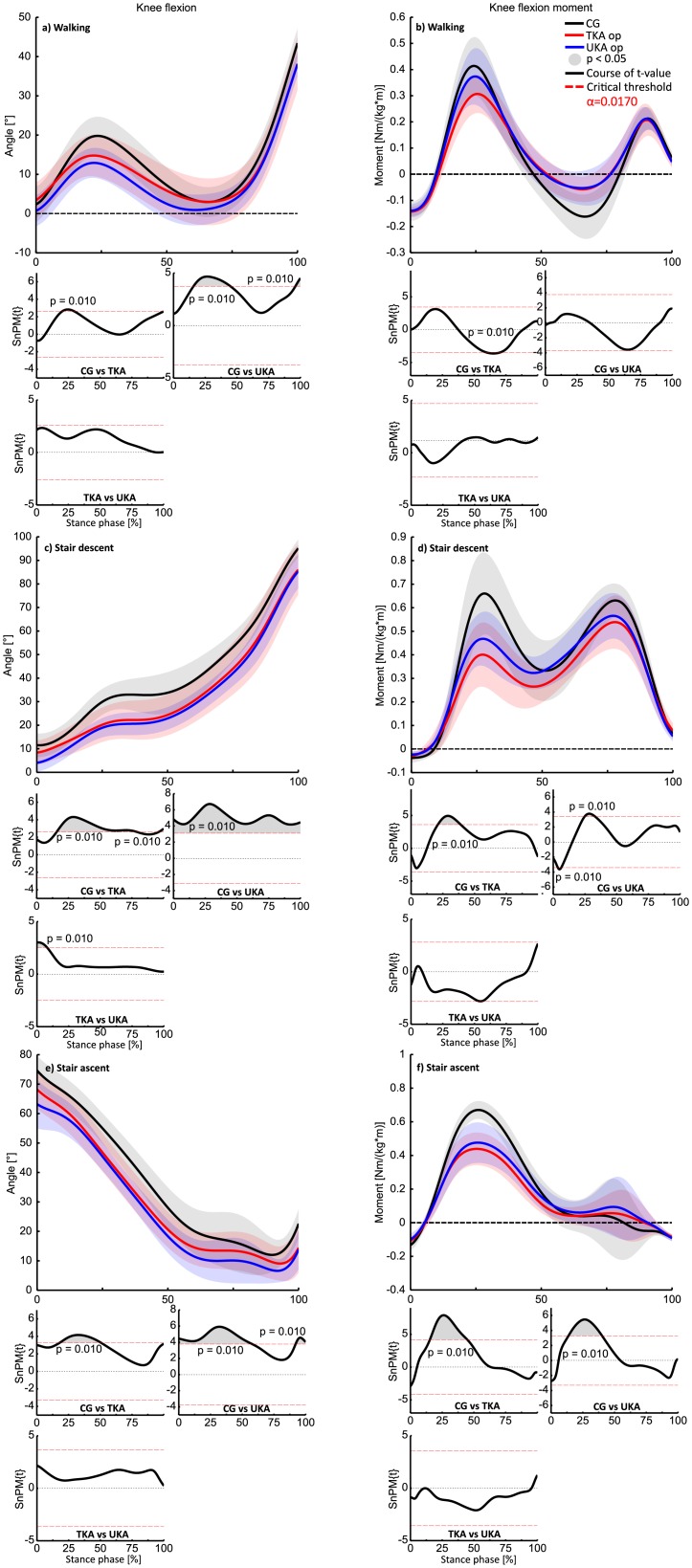
Sagittal plane kinematics and kinetics group comparisons during walking (a, b), stair decent (c, d) and stair ascent (e, f). The time series with positive values indicate knee flexion angles or moments respectively and are presented as mean curves ± standard deviations (SD, shaded areas). SnPM-{t} trajectories (post -hoc comparisons between groups) are illustrated below each subfigure. If a {t} trajectory exceeds the appropriate critical threshold (α = 0.017), the null-hypothesis can be rejected. The width of the exceeded threshold characterizes the temporal extent of the null-hypothesis (suprathreshold clusters illustrated by grey shaded areas underneath the SnPM-{t} trajectories).

Irrespective of the locomotion task, both KA groups presented reduced knee flexion angles with corresponding reduced knee flexion joint moments compared to the CG (Fig 3). This impairment was mainly apparent during 15–50% of stance phase, whereby the UKA-group revealed the stiff knee pattern to a greater extent particularly during the stair descent task almost throughout the entire Stance phase (Fig 3c). No statistical significant differences were found between the TKA and UKA-group in the sagittal plane except for the stair descent task during the first 12% of stance phase. The UKA-group initiated the stance phase with less flexed knee angles compared to the TKA-group.

#### Interlimb comparison

**Fig 4:**
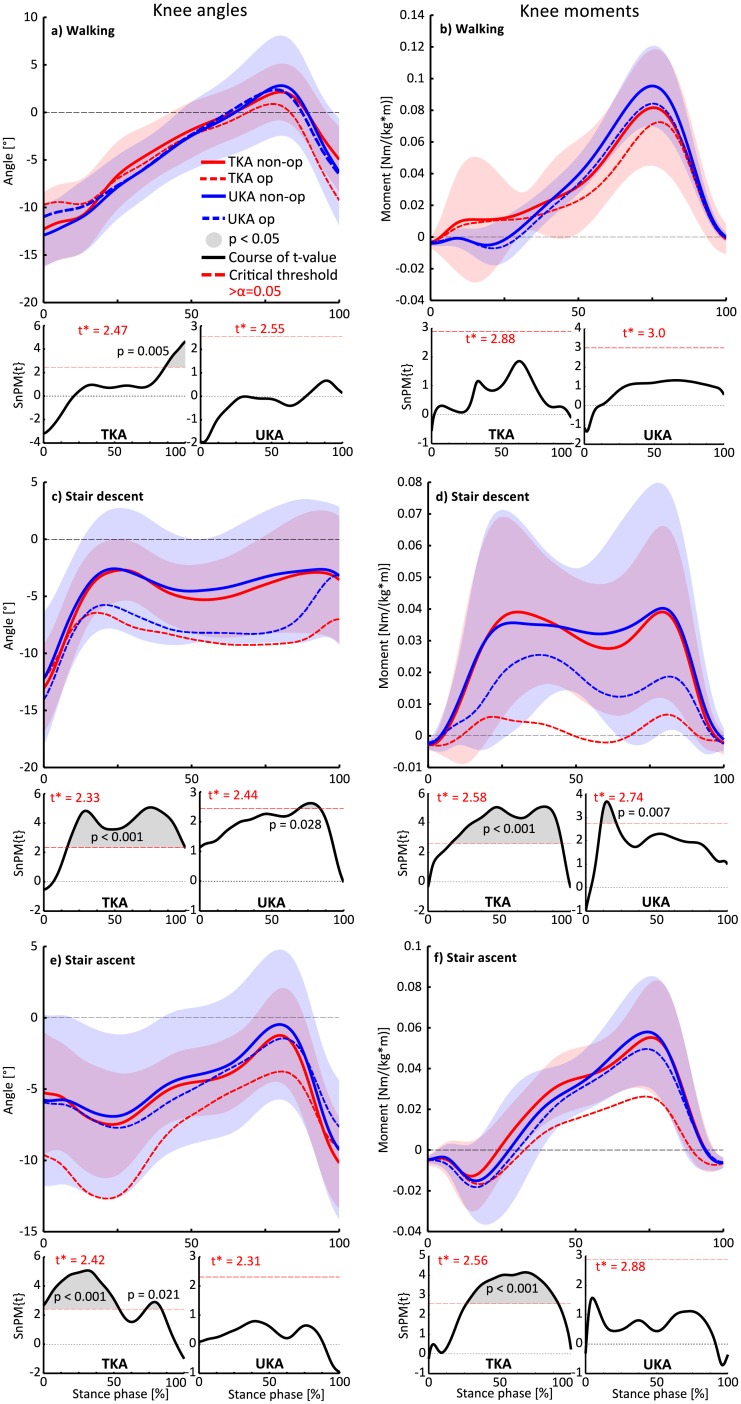
Transverse plane knee kinematics and kinetics inter limb comparisons during walking (a, b), stair descent (c, d) and stair ascent (e, f). The time series with positive values indicate internal rotation angles or moments respectively and are presented as mean curves ± standard deviations of the non-op knee (SD, shaded areas). SnPM-{t/F} trajectories (paired t-test between the op and non-op knee of the TKA and UKA-group) are illustrated below each subfigure. If a {t/F}-trajectory exceeds the appropriate critical threshold (α = 0.017), the null-hypothesis can be rejected. The width of the exceeded threshold characterizes the temporal extent of the null-hypothesis (suprathreshold clusters illustrated by grey shaded areas underneath the SnPM-{t} trajectories).

Similar to the group comparisons, the interlimb comparisons, presented in Fig 4 exhibited compromised axial rotational constraints in the replaced knee joint compared to the non-op limb in the TKA-group. Particularly during stair climbing, the internal rotation motion of the op knee was impaired apparently, since the time series progress were significantly different throughout a considerable period of the stance phase in comparison with the non-op knee. This is evident from the suprathreshold clusters, which exceeded the corresponding critical thresholds (stair descent: {t} = 2.33, 19–90% of stance, p = 0.001; stair ascent: {t} = 2.42, 0–54%, p = 0.001, 73–84%, p = 0.021) (Fig 4c and 4e).

With regard to the internal rotation moments, the TKA-group showed significantly reduced torsional joint moments throughout the major part of the stance phase (26–91%; {t} = 2.56; p = 0.001) in their op knee during stair ascent. The peak internal rotation moments values were decreased by 48% in contrast to the non-op knee.

The SnPM analysis revealed statistically significant asymmetries in the UKA-group during stair descent, including noticeable inter-individual moment variations (Fig 4d). Furthermore, the UKA-group demonstrated impaired internal rotation motion of the op knee compared to their non-op limb to the same extent as the TKA-group only during stair descent within 68–83% of stance (p = 0.028).

## Discussion

To the authors’ knowledge, SPM or SnPM as a statistical approach, has only recently been applied in analyzing biomechanical datasets obtained from patients after KA-surgery [30]. Instead of formulating specific and narrow *a priori* hypothesis, SnPM provides a non-directional hypothesis testing, which is appropriate for one-dimensional datasets used in biomechanical studies [31]. Pataky et al. [31] showed that traditional zero-dimensional statistical analysis of one-dimensional time series yield false positive results with much higher probabilities than α = 0.05. Furthermore, the extraction of scalar parameters at a specific time point is prone to type II error, because statistically significant differences may exist at other time points of the analyzed curves [32]. Hence, the aim of the current study was to analyze transverse plane kinematics and kinetics in KA-patients in various ADL considering a statistical evaluation of the entire time series. The most important finding was that the TKA-group exhibited impaired knee internal rotation motion compared with the CG (Fig 2). Although the rotational constraint was present in level walking and both stair climbing tasks, the discrepancy between TKA-group’s op knee and the CG increased considerably during stair ascent and descent. In this regard, the op knee of the TKA-group revealed asymmetric internal rotation angle pattern compared with their non-op knee, particularly during stair descent and ascent. Fig 4c clarifies that the tibia was significantly (p = 0.001) more externally rotated relative to the femur almost throughout the entire stance phase of the stair descent task. These findings are in accordance with the results of a recently published study [10]. The authors also showed impaired rotational mobility in the transverse plane of the knee joint in TKA patients, however only during decline walking. Stair climbing was not considered in the mentioned study. No statistically significant differences were detected in level walking and incline walking, probably due to the traditional zero-dimensional statistical analysis of one-dimensional time series. Similar results were reported in previous studies concerning rotationally constrained knee internal rotation only during gait using optoelectronic motion analysis in TKA patients compared to a healthy CG [33,34]. Fluoroscopic investigations showed rather heterogeneous results. On the one hand, knee internal rotation was impaired in kneeling activities [14,35–38], on the other hand, TKA patients experienced transverse plane knee kinematics similar to normal [37,39,40]. This inconsistency is likely related to the constraints of different TKA designs, including among other things posterior stabilized or cruciate ligament-retaining, mobile or fixed-bearing, single or multi-radius implants [41].

Despite the rising popularity of UKA [42,43], biomechanical investigations of UKA have been by far less conducted compared with studies including TKA. However, it is often stated that UKA provides normal function and knee kinematics, including axial rotational motion, in contrast to TKA [44–46]. Nevertheless, there is evidence that despite the preservation of the cruciate ligaments, which are stated to be essential prerequisites for normal knee kinematics [47], axial rotation is compromised in a subpopulation of UKA patients [48–51], Argenson et al. [48] proposed progressive laxity of the anterior cruciate ligament as a possible explanation. Patil et al. [46] conjectured that the articular surface geometry plays a decisive role in constraining knee internal rotation pattern in TKA. In this regard, high congruency of TKA implants can lead to an undesired restriction of axial rotation about the longitudinal axis.

Due to methodological limitations in the previously published literature, the assumption of more natural kinematics in favour of UKA cannot be regarded as proven. To date, TKA, UKA patients and a healthy CG have rarely been considered in mutual case control studies. Furthermore, in most cases unnatural kneeling activities or simple level walking have been analyzed. In a study by Jung et al. [52] similar to the current study’s transverse plane knee kinematics were reported in a TKA- and UKA-group during stair climbing, whereby the UKA-group was supposed to present knee motion closer to normal than the TKA-group. A healthy CG or the investigation of the non-op limb was not included. In the current study, the UKA-group exposed time series similar to the TKA-group in terms of compromised knee internal rotation, particularly during stair descent (Fig 2c). This result is confirmed by the significantly lower knee internal rotation time series of the UKA-group’s op knee compared with their non-op knee. Generally, these results highlight the importance of investigating physically or neuromuscular respectively more demanding ADL apart from level walking, since this task exhibited no appreciable impairments in the transverse plane. Besides, these results disprove the hypothesis that UKA provides closer to normal kinematics and kinetics.

An additional explanation for why the knee internal rotation was compromised in both KA-groups in the current study, could be a simultaneously decreased knee flexion, since the tibia rotates internally with increasing knee flexion. Reduced knee flexion angles are a common pattern in TKA in level walking as well as stair climbing [3–5]. One possible way to explain this is that due to the resection of the ACL patients attempt to reduce knee flexion by an increased, prolonged and unconscious innervation of the hamstring or gastrocnemius muscles in the sense of high levels of co-contraction. This neuromuscular abnormal activation reduces the anterior tibial translation, induced by the quadriceps muscle, and the tibial internal rotation [53–56], substituting the function of ACL [57]. The sagittal plane results of the current study (Fig 3)) illustrate that both KA-groups accomplished the investigated locomotion tasks with reduced knee flexion angles compared with the CG. Surprisingly, the UKA-group exposed more considerable impairments than the TKA-group throughout an appreciable period of the stance phase during stair climbing (Fig 3c and 3e). This abnormal pattern of KA-patients is probably related to the reduced quadriceps strength in the op knee [6] as well as to the theory of ACL-insufficiency in UKA patients proposed by Argenson et al. [48]. Wolterbeek et al. [14] associated high levels of co-contraction and reduced knee transverse rotation with large early migrations, i.e. in TKA patients during a step up task. This aspect has a significant clinical relevance in term of prosthetic loosening. Surely, further KA studies are needed including electromyographic measurements in order to quantify co-contraction of the knee extensor and flexor muscles and its influence on inhibited knee internal rotation, especially in various ADL involving high knee flexion angles.

In terms of the torsional moments, level walking revealed the highest peak moment values of all included locomotion tasks (Fig 2b). This result is in accordance with previous published data by Bergmann et al. [8]. The torsional moments during gait, measured by means of instrumented knee endoprosthesis, even exceeded the values during jogging. Moreover, the authors highlighted the large interindividual variation of the transverse joint force and joint moment components, particularly in non-cyclic activities like stair descent, which is consistent with the current study. The high standard deviations, illustrated by the shaded areas in Fig 2d, demonstrate the pronounced interindividual variation during stair descent. However, it should be considered that the large interindividual variation in each group of the current study may result from the relative short lever arm in the transverse plane. Hence, marginal force vector alterations in the transverse plane can change the knee moment into an external or internal moment respectively. Due to the above mentioned issues, statistically significant intergroup (e.g. TKA vs UKA) and interlimb (e.g. UKA op knee vs UKA non-op knee) differences should be treated with caution considering the stair descent negotiation.

Aside from the above mentioned possible explanations for the impaired transverse plane knee motion in KA-patients, a further explanatory approach could be a combination of high friction and relatively low torsional moments. The authors hypothesize that decreased internal rotation moment magnitudes during stair climbing along with increased friction in artificial knee joints might reduce axial rotation which is therefore more apparent in ADL involving high normal forces. This mechanism could explain why the UKA-group also tended to perform stair climbing with reduced knee internal rotation, even though the tibia inlays were flat in this group. Hence, high congruency of the implant components should not lead to restricted axial knee motion in UKA designs with flat tibial inlays. In this context, Kraemer et al. [13] stated that the magnitudes of the moments transmitted by the tibial plateau to the bone-implant interface should be proportional to the force of friction transmitted, aside from the congruency of the implants, by the articular surface. Due to the torsional shear, enhanced by high friction, acting on the bone-implant interface the authors reported axial migration of the tibia plateau [13,14,58]. Excessive early migration can lead to aseptic loosening of the tibial component which is currently the major, but least understood reason of failure in UKA and TKA [59,60]. The results of the current study indicate that of all investigated ADL, level walking in combination with relatively high internal rotation moments, high friction and approximately 640 000 step cycles per year [61] could be a decisive ADL in terms of accelerated torsional tibial implant migration.

This study includes several limitations. Transverse plane time series should be interpreted with caution, since measurement and modelling issues could bias the results [62]. Thus, a special effort has been made to include only subjects with BMI values lower than 31 (Ø BMI: TKA-group = 27.4, UKA-group = 27.4) with the aim to reduce soft tissue artifacts. Nevertheless, both KA-groups had statistically significant higher BMI values compared to the CG, which should be taken into account when interpreting the results. Additionally, the knee joint was modelled as a spherical joint in order to calculated more realistic joint kinematics. The groups were not gender-matched, which may have affected the results of this study. On account of missing force platforms during level walking in the ARCUS Clinics, ground reaction forces were not measured. Hence, only eleven TKA and thirteen UKA subjects were considered for statistical analysis of the knee internal rotation moments. Preserving or substituting of the posterior cruciate ligament might have an effect on axial rotation of the replaced knee joint [63,64]. Although there was no difference between the TKA-CR and TKA-PS group, conclusions should be drawn with caution considering the impaired knee internal rotation in the TKA-group. The lack of differences between both TKA implant designs may be due to the low statistical power as a result of the small sample size, particularly in the TKA-CR group, failing to reject the null-hypothesis. On the other hand, the magnitude of the differences between both groups are presumably clinically not relevant during stair climbing regarding the transverse plane kinematics (S1D and S1F Fig).

## Conclusion

If the common indications for a UKA described in Kozinn and Scott [65] and Hurst and Berend [66] are fulfilled, a surgeon has to balance the risks and opportunities of the relevant knee arthroplasty type and make an appropriate choice. The results of the current study considering transverse plane kinematics and kinetics revealed that the TKA-group presented restricted knee internal rotation during all investigated ADL. The abnormal rotational pattern was more apparent during stair climbing than in level walking. The SnPM analysis depicted similar compromised knee internal rotation time series of the UKA-group to the TKA-group and a significant asymmetry between the op and non-op knee particularly during stair descent. Conclusively, this underlines that appreciable advantages in terms of ‘more normal’ transverse plane kinematics and kinetics of the UKA-group compared to the TKA-group could not be shown in this study. Aside from the common reasons, which can be responsible for the rotational deficit, the authors propose friction and co-contraction as potential aspects contributing to the inhibited knee internal rotation. Certainly, further biomechanical studies are needed to prove the theory of beneficial kinematics of UKA over TKA, including both arthroplasty types as well as a healthy CG in the analysis. The acquired comprehensive understanding of possible biomechanical differences or similarities will help surgeons in the decision-making process.

## Supporting information

S1 Fig Sagittal and transverse plane knee kinematics comparison between the TKA-CR and TKA-PS group during walking (a, b), stair descent (c, d) and stair ascent (e, f). The time series with positive values indicate knee flexion or internal rotation angles respectively and are presented as mean curves ± standard deviations (SD, shaded areas). SnPM-{t} trajectories (post-hoc comparisons between TKA-CR versus TKA-PS) are illustrated below each subfigure. If a {t}-trajectory exceeds the appropriate critical threshold, the null-hypothesis can be rejected.
(PDF)

S2 Fig Transverse plane knee kinematics and kinetics SnPM post-hoc comparison during walking, stair descent and stair ascent. If a {t}-trajectory exceeds the appropriate adjusted critical threshold (α = 0.017), the null-hypothesis can be rejected. The width of the exceeded threshold characterizes the temporal extent of the null-hypothesis (suprathreshold clusters illustrated by grey shaded areas underneath the SnPM-{t}-trahectories).
(PDF)
