# Supplementary figures and images for: Compromised knee internal rotation in total knee arthroplasty patients during stair climbing
Source: PLoS One. 2018 Oct 10;13(10):e0205492. doi: 10.1371/journal.pone.0205492 (PMC6179266; doi:10.1371/journal.pone.0205492)

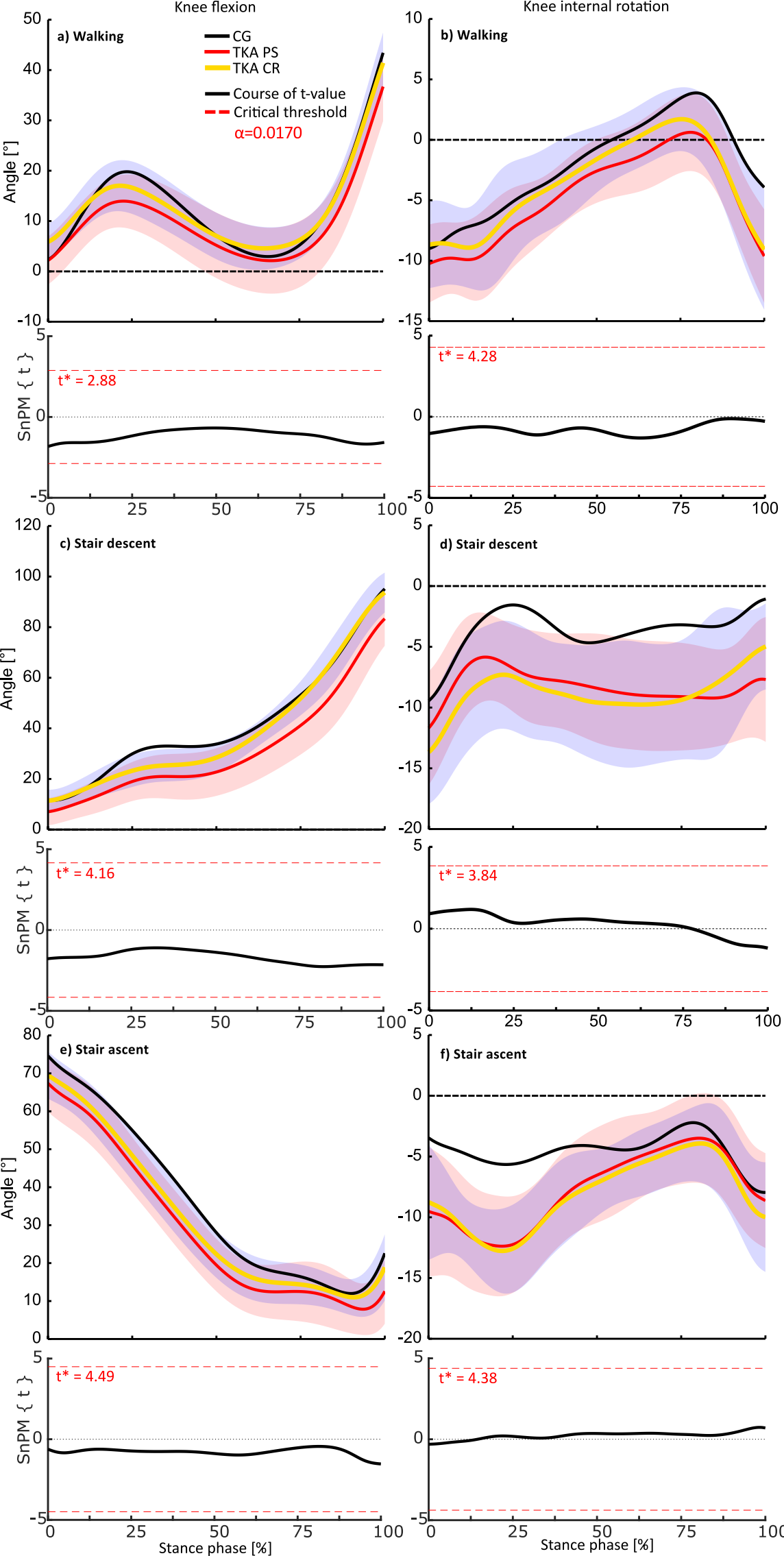

Supplement: S1 Fig — The time series with positive values indicate knee flexion or internal rotation angles respectively and are presented as mean curves ± standard deviations (SD, shaded areas). SnPM-{t} trajectories (post-hoc comparisons between TKA-CR versus TKA-PS) are illustrated below each subfigure. If a {t}-trajectory exceeds the appropriate critical threshold, the null-hypothesis can be rejected. (PDF) [file pone.0205492.s001.pdf]
