# Supplementary material for: Compromised knee internal rotation in total knee arthroplasty patients during stair climbing
Source: PLoS One. 2018 Oct 10;13(10):e0205492. doi: 10.1371/journal.pone.0205492 (PMC6179266; doi:10.1371/journal.pone.0205492)
Supplement: S2 Fig — If a {t}-trajectory exceeds the appropriate adjusted critical threshold (α = 0.017), the null-hypothesis can be rejected. The width of the exceeded threshold characterizes the temporal extent of the null-hypothesis (suprathreshold clusters illustrated by grey shaded areas underneath the SnPM-{t}-trahectories). (PDF) [file pone.0205492.s002.pdf]

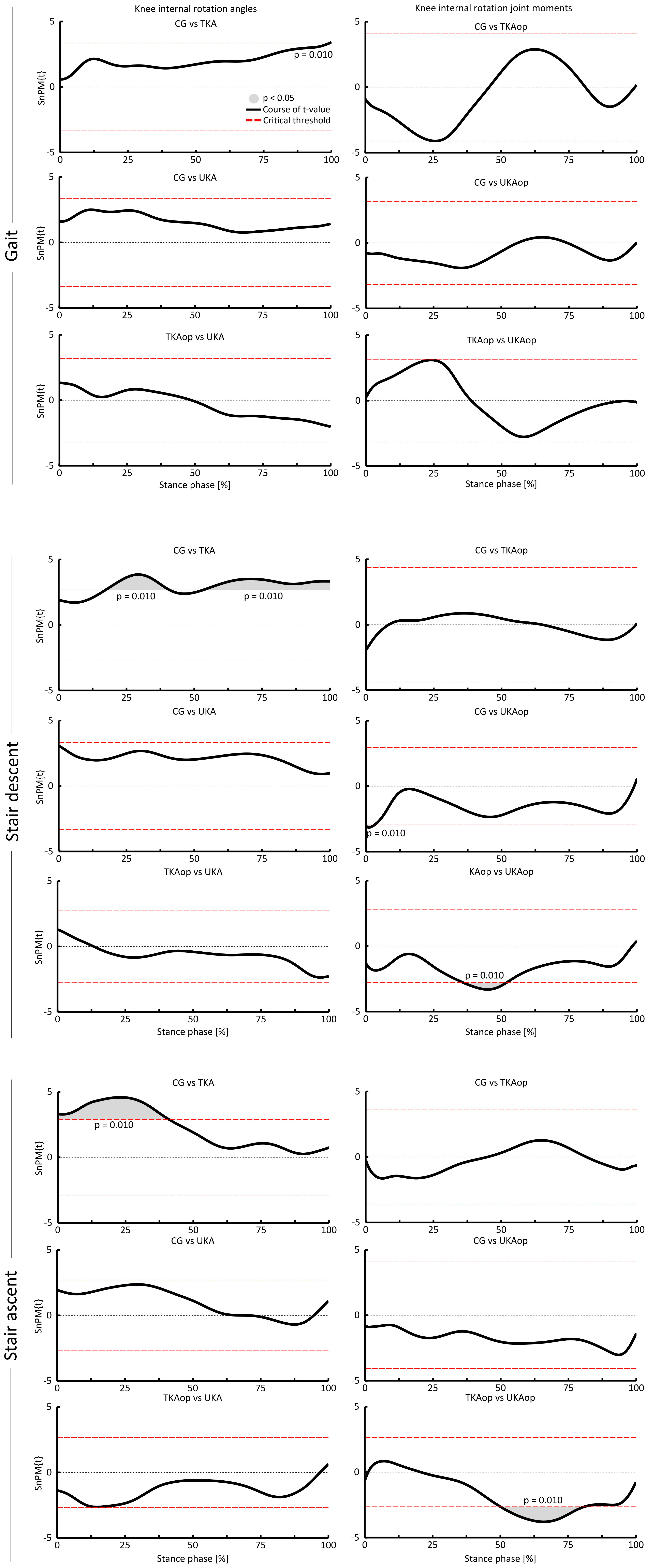

S2 Fig 1. Transverse plane knee kinematics and kinetics SnPM post-hoc comparisons during walking, stair descent and stair ascent.

If a {t}-trajectory exceeds the appropriate adjusted critical threshold (α = 0.017), the null-hypothesis can be rejected. The width of the exceeded threshold characterizes the temporal extent of the null-hypothesis (suprathreshold clusters illustrated by grey shaded areas underneath the SnPM-{t}-trajectories).
